# Enzymatic production of xylooligosaccharide from date (*Phoenix dactylifera* L.) seed

**DOI:** 10.1002/fsn3.1964

**Published:** 2020-11-03

**Authors:** Davoud Ataei, Zohreh hamidi‐Esfahani, Hassan Ahmadi‐Gavlighi

**Affiliations:** ^1^ Department of Food Science and Technology Faculty of Agriculture Tarbiat Modares University Tehran Iran

**Keywords:** date seed, enzymatic production, hydrolysis conditions, potential prebiotic, xylooligosaccharide

## Abstract

Date palm (*phonix dactylifera L*.) is an important tropical fruit growing in central and southern regions of Iran. Date seed is composed of cellulose, hemicellulose, and lignin, that make it an excellent candidate for xylooligosaccharide (XOS) production. In this study, two different protocols are used for the extraction of hemicellulose from date seeds. In the first protocol, hemicellulose (xylan1) was extracted by 2.25 M alkaline solution at room temperature for 24 hr. In the second protocol, date seed was treated with LCHTA (low concentration, 0.1 M, high temperature, 80°C, alkaline solution) for 3 hr, and thereafter, hemicellulose (xylan2) was extracted by 2.25 M alkaline solution at room temperature for 24 hr. The carbohydrate units of xylan1 and xylan2 were qualified and quantified by HPAEC‐ PAD. Side groups of xylan1 and xylan2 were detected by FTIR. In the next step, xylan1 and xylan2 were exposed to two commercial endoxylanases namely veron 191 and pentopan mono BG. Temperature, pH, time, and enzyme dosage of hydrolyzation were optimized to maximize XOS and minimize xylose. The results showed that the enzymes successfully hydrolyzed xylan2 and produced XOS, but cannot hydrolyze xylan1. Pentopan mono BG and veron 191 produced the highest amount of XOS after 4 (1.17 mmol/g) and 6 hr (1.13 mmol/g) of incubation, respectively. Conversion factors of xylan2 to XOS for pentopan mono BG and veron were 0.41 and 0.36, respectively. This study presence the possible prebiotic properties of date seed XOS and its application in functional foods.

## Graphical abstract

Low polymerization degree xylooligosaccharides (XOS) were produced from date seed. A special treatment (0.1 N NaOH at 80°C) is essential prior to xylan extraction. Temperature, pH, reaction time and enzyme dosage were optimized. Xylanase type, enzyme dosage and hydrolysis period affect the degree of polymerization of XOS.

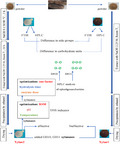

## 1 INTRODUCTION

The use of agricultural by‐products as raw materials in order to produce valuable products can also lead to reducing wastes and production costs. Date palm is an important agricultural product in Iran that a large amount of it, especially low‐grade dates, is used in date syrup production, date chocolate, vinegar, and liquid sugar. The seeds are by‐products from date industry and consist of 10%–15% of fruit weight that are rich in phenolic compounds with antioxidant activities (Al‐farsi et al., 2007). Date seed contains 7%–10% oil which makes it a suitable source for the production of edible oil or antioxidants (Ashraf & Hamidi‐Esfahani, 2011).

Moreover, the date seed is rich in dietary fibers (77%–80%) such as hemicelluloses that is composed of heteropolymers including xylans, arabinans, and mannans (Al‐Farsi & Lee, 2008). Xylan is the main hemicellulose polymer in cereals and hardwoods (Vazquez et al., 2006) consisting of β‐1,4‐linked D‐xylose backbone with different side chains like L‐arabinose, D‐galactose, acetyl group, and galacturonic residue (Kiran et al., 2013). Hydrolysis of xylan releases xylose and xylooligosaccharides (XOS) with different polymerization degrees (DP) (Falk et al., 2014). Several studies have reported prebiotic properties of XOS which accelerates the population growth of intestinal probiotic bacteria and makes it useful for production of functional foods (Gullon et al., 2011).

Different methods have been suggested for XOS production such as the autohydrolysis of lignocellulosic materials that is a hydrothermal process at high temperatures (>130°C) or xylan extraction from lignocellulosic material at mild temperature and pH conditions followed by acidic hydrolyzation. However, undesirable compounds such as monosaccharides and furfural are produced in these methods which must be removed by subsequent processes. Therefore, the production costs increase, and on the other hand, there is no precise control over the final product (Carvalho et al., 2013). Enzymatic hydrolysis as a novel extraction technique is considered as the best method to produce XOS with food industry grade (Carvalho et al., 2013), and the use of enzymes causes the desired products with high purity (Gowdhaman & Ponnusami, 2015).

Xylan hydrolyzing enzymes have xylosidase or xylanase activity. Xylosidases release xylose from the backbone of xylan, while xylanases release XOSs from the bone of xylan (Polaina & MacCabe, 2007). Xylanases belong to the two main families of carbohydrate‐hydrolyzing enzymes (GH10 and GH11). Several researchers have reported that GH10 and GH11 families produce low DP and high DP XOS, respectively (Kiran et al., 2013). In commercial processes, the operational conditions must be optimized for high productivity. According to the definition, the productivity of an enzymatic process equals the weight of the product(s) per unit weight of the enzyme at the defined operation conditions such as pH and temperature (Amit et al., 2018).

Use of commercial enzymes reduces the cost of hydrolyzation. The source of xylanase, pretreatment of seed, and xylan composition are the main factors influencing the XOS yield (Carvalho et al., 2013). Alkaline extraction is the best option for hemicellulose extraction which that the concentration and temperature of alkaline solutions have a direct impact on hemicellulose yield and structure (Jayapal et al., 2013). So far, several studies have been carried out addressing the XOS production from several agricultural by‐products such as wheat and rice bran (Wang et al., 2010), corncob (Chapla et al., 2012), cane bagasse (Brienzo et al., 2010), olive stone (Nabarlatz et al., 2007), and stalks (Akpinar et al., 2007, 2010; Samanta et al., 2013; Wang et al., 2018).

To the author's knowledge, there is no report on date seeds. Therefore, the aims of this study were to extract hemicellulose from date seeds by two different extraction techniques and compare two commercial xylanases (veron 191 from GH10 and pentopan mono BG from GH11 family) in order to hydrolysis of date seed hemicellulose. Moreover, the effects of pH and temperature of the enzyme processes were optimized using response surface method (RSM) and the effect of enzyme dosage and hydrolysis time was studied to maximize and minimize XOS and xylose, respectively.

## 2 MATERIALS AND METHODS

### 2.1 Date seed

Date seeds (Kabkab variety) were obtained from a local date factory in Behbahan, Khuzestan, Iran, then washed, and dried under sun exposure. Afterward, the seeds were grounded by a shear miller (Ahrar mill company) and particles smaller than 2 mm were separated by sieve and stored at −20°C prior experiments.

### 2.2 Chemicals

Chemical materials with analytical grade were purchased from Merck. Birchwood xylan and sugar standards were purchased from Sigma (Deisenhofen), and XOS standards obtained from Megazyme.

### 2.3 Date seed hemicellulose extraction

**FIGURE 1:**
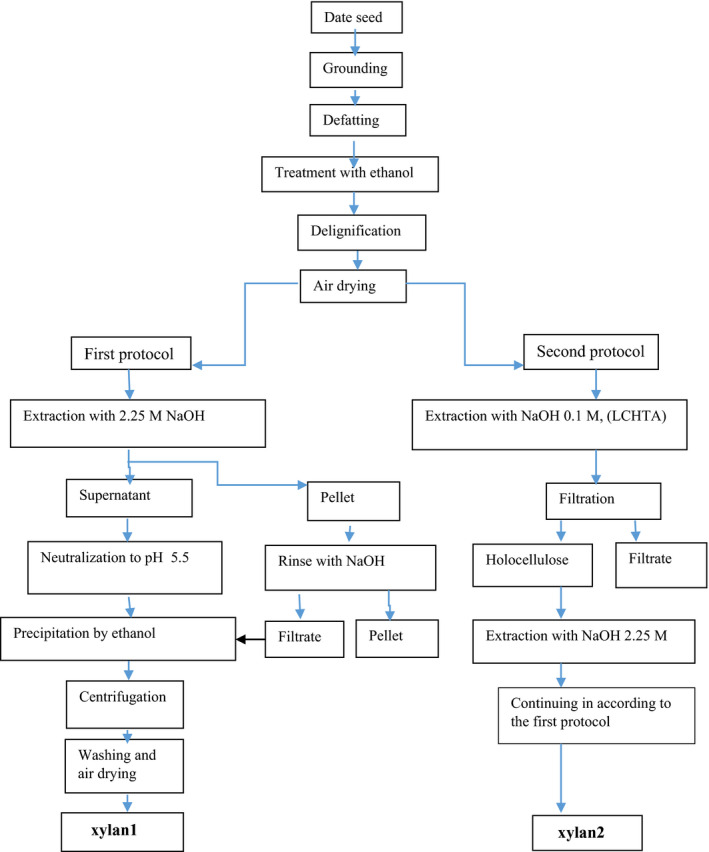
Flowchart for extraction of date seed hemicellulose (xylan1 and xylan2)

Alkali method was used for hemicellulose extraction due to its practical advantages (Liu et al., 2016). Fats, soluble sugars, and lignin were removed prior to the extraction process by the method proposed by Ishurd et al. (2003). Seed powder (30 g) was defatted for 4 hr at 40°C with hexane (1:15) and washed with solvent several times. The remaining material was added in ethanol at 75°C (1:15 w/v) for 4 hr and constantly stirred to separate monomers and colorants. In the next step, the remaining material was placed in % 0.7 sodium chloride solution (1:55 w/v) with pH 4 at 75°C, 5 hr for delignification, and then dried by hot air at 60°C. Finally, the produced holocellulose material was extracted according to the two above mentioned protocols (Figure 1):

According to the first protocol, holocellulose material was placed in 2.25 M sodium hydroxide (1:10 w/v) at room temperature (25°C) for 24 hr under nitrogen atmosphere and agitation; then, it was centrifuged. The remaining material was washed with alkaline solution, and the filtrate was added to the supernatant, and then, the total solution was neutralized to pH 5.5 with acetic acid glacial. The neutralized solution was mixed with three volumes of ethanol 95%, stored overnight at 4°C, and centrifuged at 8,500 g. The hemicellulose (xylan1) was washed several times with ethanol and dried in hot air at 60°C.

As for the second protocol, LCHTA (low concentration and high‐temperature alkaline) treatment was done based on the method described by Chen et al (2012) before the extraction of hemicellulose. Accordingly, holocellulose material was exposed to dilute solution NaOH (0.1 M) at 80°C for 3 hr. Afterward, hemicellulose (xylan2) was extracted in accordance with the first protocol.

### 2.4 Sample preparation for HPAEC–PAD

To identify of structural carbohydrate units, the procedure was performed according to the method by Balaghi et al. (2011). Two mL of trifluoroacetic acid (TFA) 2 M with 20 mg xylan1 or xylan2 powder was mixed in glass vials at 121°C for 2 hr. Afterward, the vials were maintained overnight in order to eliminate TFA and then lyophilized (Scanvac Coolsafe™, Labogene Company), and stored at −20°C prior analysis.

### 2.5 Qualitative identification of carbohydrate units of xylan1 and xylan2

Carbohydrate units of the hemicellulases (xylan1 and xylan2) were identified by a BioLC system consisting of GS50 gradient pump, ED50 electrochemical detector, and AS50 chromatograph connected to AS50 autosampler. Separations were done by an analytical column of CarboPac PA1 (Thermo Fisher Scientific Inc., 4.6 × 250 mm) according to the method by Balaghi et al. (2011) with slight changes. The eluent flow rate was set on 1 ml/min. A two‐eluent system comprising of deionized water (18.2 mU, 25°C) and 500 mM NaOH solution was used for monosaccharide separation. Neutral monosaccharides were eluted isocratically by 15 mmol NaOH solution for 15 min followed by second isocratic elution at high concentration NaOH (500 mmol) for 10 min to elute uronic acids. Before each injection, column re‐equilibration program was run for 5 min with 15 mmol NaOH. Before analysis, hydrolysates of xylan1 and xylan2 were resolved in double deionized water and was brought to 5 ml, then filtered by 0.22 µM syringe filter, and used for injection into HPAEC–PAD. The carbohydrate standards (fucose, rhamnose, arabinose, galactose, glucose, xylose, galacturonic, and glucuronic acid) were prepared at 1, 2, 3, 4, 5, 6, 7, 8, 10, 12, 14, and 15 ppm concentrations and injected in HPAEC. Standard curves were drawn by Excel 2016 software based on chromatograms surface and concentration for every sugar.

### 2.6 Fourier transform infrared (FTIR) spectroscopy

Fourier transform infrared spectra of xylan1 and xylan2 analyzed by an FTIR spectrophotometer (PerkinElmer‐Frontier) at 4,000–400 cm^−1^ and spectral range were obtained for the identification of side groups of the hydrolysates. Briefly, 1.5 mg xylan1 or xylan2 was mixed with 170 mg KBr and put under pressure for access to the tablets with the same thickness (Jayapal et al., 2013).

### 2.7 Measurement of lignin

Soluble and insoluble lignin contents of date seeds, xylan1, and xylan2 were measured using the method introduced by Sluiter et al. (2012).

### 2.8 Xylanase activity measurement

Two commercial xylanases veron 191 from *Aspergillus niger* (GH10 family) and the recombinant pentopan mono BG expressed in *Aspergillus Oryzae* (GH11 family) were purchased from AB Enzyme and Sigma‐Aldrich, respectively. Activities of the two commercial xylanases were measured using 2% birchwood xylan solution as the substrate according to the method by Bailey et al. (1992).

For both enzymes, several runs at different times (10, 20, 30 min) were carried out in microtubes on the thermo‐shaker (Bioer‐MB102, China). For each run, 1 ml xylan solution, 0.9 ml acetate buffer, and 0.1 ml enzyme solution were mixed (pH 5, T = 50°C for pentopan and T = 40°C for veron). The reactions were stopped at 95℃. DNS method (Miller, 1959) was used for the determination of reducing sugar released during the enzymatic reaction in which one unit is defined as the amount of enzyme that liberates 1.0 µmol of xylose per min at 40 or 50°C and pH 5 (Kiran et al., 2013).

### 2.9 Optimization of pH and temperature for xylan2 hydrolysis

pH and temperature of the hydrolysis reaction of xylan2 were optimized with the response surface method. Central composite design (CCD) in triplicate was used by Minitab software (version 16). In doing so, the numbers of runs and center points were chosen at thirteen and five, respectively. Alpha (α) for all replications was set on 1.414. For both enzymes, pH level (X1) within 4.5–5.8 with a boundary of 4.2–6.1 (±α) was used in the experimental design. For veron 191, the temperature level (X2) within 35–45°C with a boundary of 32.9–47 (±α) and for pentopan mono BG, X2 45–65°C with a boundary of 40.8–69.1°C (±α) were used. For all runs, reaction time within and enzyme dosage were 60 min and 0.3 U/ml, respectively. Reactions were carried out in microtubes in thermo‐shaker (0.5 ml %2 xylan +0.4 ml buffer +0.1 ml of enzyme solution). At the end of each run, the enzyme was deactivated at 95℃ and microtube centrifuged at 8500 g for 10 min. The supernatant (including hydrolysates) was separated and mixed with DNS solution. After 15 min at 95°C, the samples absorption was read at 540 nm by a spectrophotometer (Agilent‐ Carry60). Several concentrations of pure xylose solutions as standards were used to plot the standard curve (Chapla et al., 2012).

### 2.10 Hydrolysis of xylan2 by the enzymes under optimum conditions

Xylan2 was hydrolyzed by the enzymes at the optimum pH and temperature. Reaction time and enzyme dosage were optimized using the statistical method of one‐factor‐at‐a‐time to obtain a higher amount of XOS. Initially, hydrolysis time was fixed on 60 min and enzyme dosage was selected as 10, 20, 30, and 40 U/g xylan2. In the next step, the enzyme dosage was fixed on 20 U/g and hydrolysis time was selected 60, 120, 240,360, and 480 min for pentopan mono BG and 120, 240, 360, 480, and 900 min for veron. For each run, the reaction volume was 1 ml (0.5 ml xylan 2% + buffer + enzyme solution), and the microtubes were placed in thermo‐shaker, and after the reaction termination, enzyme inactivation was done at 95°C and the hydrolysates centrifuged at 8500 g.

### 2.11 Detection of hydrolysates of xylan2

Hydrolysates of xylan2 were analyzed by the HPAEC–PAD system that used to detect hemicellulose composition according to the method by Rantanen et al. (2007) with a few modifications. HPLC grade water, sodium hydroxide (200 mmol), and sodium acetate (500 mmol) were used as the mobile phase system with gradient eluent and a total time of program 46 min: 0–6 min NaOH 50 mmol, 6–16 min NaOH 42 mmol and sodium acetate 50 mmol, 16–26 min NaOH 50 mmol and sodium acetate 100 mmol, 26–36 min NaOH 200 mmol (to wash XOS), and 36–46 min NaOH 50 mmol (to equilibrate the column for the next injection) at 30°C. All the solutions and water were degassed with nitrogen gas. Standard solutions of X1 (xylose), X_2_ (xylobiose), X_3_ (xylotriose), X_4_ (xylotetraose), X_5_ (xylopentaose), and X_6_ (xylohexaose) were prepared at 1, 2.5, 4, 5, 6, 8, and 10 ppm concentrations and injected into HPAEC. The curves of standards were drawn by Excel software.

## 3 RESULTS AND DISCUSSION

### 3.1 Hemicellulose and carbohydrate contents of date seed

The mean contents of xylan1 and xylan2 in date seeds were 14% and 13.2%, respectively. Ishurd et al. (2003) reported a higher hemicellulose content of *Phoenix dactylifera* L. date seeds (25%– 26%). The carbohydrate composition of xylan1 and xylan2 is shown in Table 1. Xylan is the most common sugar in date seeds hemicellulose. Amounts of arabinose and galactose in seeds were 0.015 and 0.35 mmol/g xylan1, respectively. However, the amount of galactose in xylan2 was 0.06 mmol/g of while with no arabinose was detected. No glucose, mannose, and uronic acids were detected in both xylans. Ishurd et al. (2003) analyzed the carbohydrate composition of hemicellulose from date seeds and reported that the contents of xylose, uronic acid, arabinose, galactose, glucose, and mannose were 72.2, 12.1, 4.4, 2.8, 5, and 2.8 mol%, respectively.

**TABLE 1:** Carbohydrates composition analysis of xylan1 and xylan2 (mmol/g)

	Uronic acid	Glucose	Galactose	Arabinose	Xylose
Xylan1	ND	ND	0.35 ± 0.004	0.015 ± 0.001	5.29 ± 0.03
Xylan2	ND	ND	0.06 ± 0.005	ND	5.49 ± 0.02

Note

Data represents the mean ± standard deviation of triplicate analysis.

Abbreviation: ND, Not Detect

**FIGURE 2:**
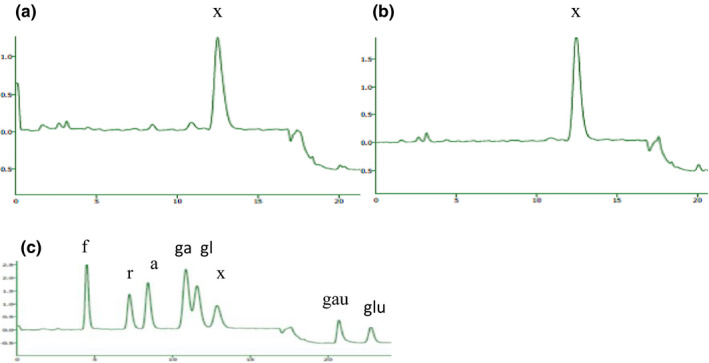
HPAEC–PAD chromatograms of thermochemical hydrolysates of (a) xylan1, (b) xylan2, and (c) sugar standards: f, fucose; r, rhamnose; a, arabinose; x, xylose; ga, galactose; gl, glucose; gau, galacturonic acid; glu, glucuronic acid

A higher content of xylose was observed in xylan2 compared to xylan1 (5.29 mmol/g) in which it can be explained by the removal of side groups at high temperature (Figure 2). Chen et al. (2012) reported that LCHTA treatment (80°C) caused the deacetylation up to 75% in some cellulose materials. Moreover, Song et al. (2008) removed all acetyl groups from spruce wood by the weak alkaline solution at 70°C. In LCHTA treatment and due to the low concentration of the alkaline solution, xylan cannot be dissolved completely (Pomeranzand Meloan, 1994). The use of high temperature or high concentrations of acid or alkaline solutions due to the corrosion effect has abstained in the industrial process (Galindo‐Luna et al., 2018). Therefore, the low concentration (0.1 M) of alkaline solution and a high temperature (80°C) applied in this study to eliminate the side groups.

The amount of acid‐soluble lignin (%ASL), acid‐insoluble lignin (%AIL), and total lignin of xylan1, xylan2, and date seeds is shown in Table 2. As can be seen, total lignin contents for xylan1, xylan2, and the seeds were 6.56, 3.43, and 1.09%, respectively (*p* ≤ .05). These results suggest that sodium chlorite does not delignificate date seeds completely and a more efficient delignification observed by LCHTA treatment.

**TABLE 2:** Acid‐soluble lignin (% ASI), acid‐insoluble lignin (% AIL), and total lignin contents from date seeds, xylan1, and xylan2

	Total lignin	% ASL	% AIL
Date seed	6.56 ± 0.2^a^	2.1 ± 0.1^a^	4.55 ± 0.1^a^
Xylan1	3.43 ± 0.1^b^	0.48 ± 0.04^b^	2.95 ± 0.1^b^
Xylan2	1.09 ± 0.05^c^	0.21 ± 0.01^c^	0.88 ± 0.1^c^

Note

Data represent the mean ± standard deviation of triplicate analysis. Different superscript alphabets within the same column represent significant difference at *p* ≤ .05.

### 3.2 Fourier transform infrared (FTIR) spectroscopy of date seed xylan

**FIGURE 3:**
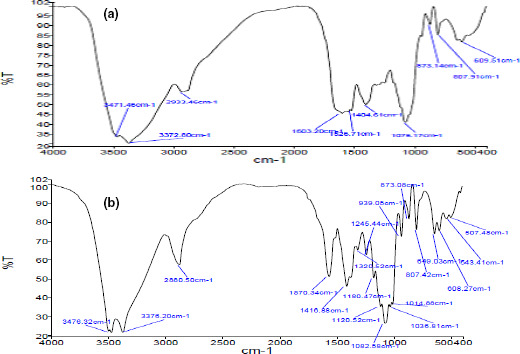
FTIR spectra of (a) xylan1, and (b) xylan2

Fourier transform infrared spectra of xylan1 and xylan2 are shown in Figure 3. Xylan1 exhibited a wide absorption band at 1590–1550 cm^−1^ that it is associated with carbonyl groups consisting of aldehydes, ketones, esters, and carboxylic acids. Meanwhile, a sharp absorption at 1,570 cm^−1^ observed for xylan2. Xylan1 showed absorption at about 1,700 cm^−1^ belonging to ester bonds (Ch'ng et al., ), while no absorption in this region for xylan2 observed. Xylan1 had an absorption band at about 1,600 cm^−1^ corresponding to OH of esters and aromatic compounds, while xylan2 showed no absorption in this region. Moreover, xylan1 had an absorption band at 1526 cm^−1^ corresponding to the aromatic ring of lignin (Jayapal et al., 2013) but xylan2 showed no absorption. These observations indicate that LCHTA treatment eliminated most of the side groups.

### 3.3 Optimization of pH and temperature for xylan2 hydrolyzing

Preliminary studies showed that the two enzymes used were not efficient enough to hydrolyze xylan1 but effectively hydrolyzed xylan2. Moreover, pH and temperature were optimized in this research as they are the main physical factors affecting the enzymes activity (Polaina & MacCabe, 2007).

Analysis of variances (ANOVAs) for xylan2 hydrolyzing of both enzymes showed that the “lack of fits” were not statistically significant (*p* > .05) (data not shown). The *p* values for the models were less than 0.001, indicating that the models were significant and could be used in monitoring the optimization. Quality of the models could be checked by using various criteria. The coefficients of determination (*R* ^2^) were .9247 and .9159 for veron 191 and pentopan mono BG, respectively. The *R* ^2^ value should lie in between 0 and 1. The closer *R* ^2^ value to 1.0 is a strong model to predict the response. As it is shown below, the CCD recommended a quadratic equation for the yields of reducing sugar for both enzymes (Y):

Veron 191: Y = −355.6 + 7.4X_1_ + 92.1X_2_−0.07$X_{1}^{2}$−7.6$X_{2}^{2}$−0.35X_1_X_2_
Pentopan mono BG: Y = −1264 + 12.44X_1_ + 389X_2_−0.1$X_{1}^{2}$−36.46$X_{2}^{2}$−0.3X_1_X_2_

Based on the above equations, the maximum yield for veron 191 was obtained at the temperature and pH of 38.2℃ and 5.15, respectively. For pentopan mono BG, the optimum temperature and pH were 53.4℃ and 5.1, respectively. To evaluate the above findings, an assay was performed under the optimal conditions for each enzyme. The predicted and experimental responses for pentopan mono BG and veron are presented in Table 3.

**TABLE 3:** Predicted and experimental values for optimization of pH and temperature for pentopan mono GB and veron 191 enzymes

Enzyme	Predicted value	Experimental value
Veron 191	29.58	30.43
pentopan mono BG	69.04	69.8

### 3.4 Hydrolysates of xylan2

Tables 4, 5, 6 depict the effect of hydrolyzation time and dosage of pentopan mono BG and veron 191 enzymes on xylan2 (XOS) production. For pentopan mono BG, the highest value of XOS was obtained within 4 hr by 20 U/g that is equal to 1.17 (X_2_ + X_3_) mmol/g which is equivalent to 0.41 g XOS/g xylan2 with the conversion factor (CF) of 0.41. The results also showed that the amount of enzyme dosage higher than 20 (Table 6), and hydrolysis duration higher than 4 hr had a negative impact on XOS yield (due to xylose production). Regarding to veron 191, the maximum XOS (1.13 mmol/g) obtained after 360 min that it was equivalent to 0.36 g XOS/g xylan2 and the conversion factor (CF) of 0.36 (Table 5). As it is shown, the amount of enzyme dosage higher than 20, and the hydrolysis duration higher than 360 min, had a negative effect on XOS yield (due to xylose production). The results also disclosed that the both enzymes produced X_2_ and X_3_ from date seeds which veron 191 produced more X_2_ than X_3_, while pentopan mono BG produced higher amounts of X_3_ than X_2_.

**TABLE 4:** XOSs (mmol/g) production by pentopan mono BG (20 U/g) during the incubation time

Hydrolysis time (hr)	1	2	4	6	8
XOS (mmol/g)	X_1_ = 0.06	X_1_ = 0.11	X_1_ = 0.13	X_1_ = 0.24	X_1_ = 0.52
	X_2_ = 0.22	X_2_ = 0.49	X_2_ = 0.55	X_2_ = 0.59	X_2_ = 0.68
	X_3_ = 0.10	X_3_ = 0.47	X_3_ = 0.62	X_3_ = 0.55	X_3_ = 0.40
Total XOS	0.32	0.96	1.17	1.14	1.08

Note

X1, X2, and X3, represent xylose, xylobiose and xylotriose, respectively.

**TABLE 5:** XOSs (mmol/g) production by veron 191 (20 U/g) during the incubation time

Hydrolysis time (hr)	2	4	6	8	15
XOS (mmol/g)	X_1_ = ND	X_1_ = ND	X_1_ = 0.13	X_1_ = 0.42	X_1_ = 0.56
	X_2_ = 0.71	X_2_ = 0.75	X_2_ = 0.82	X_2_ = 1.07	X_2_ = 0.97
	X_3_ = 0.06	X_3_ = 0.10	X_3_ = 0.31	X_3_ = 0.04	X_3_ = ND
Total XOS	0.77	0.85	1.13	1.11	0.97

Note

X1, X2, and X3 represent xylose, xylobiose, and xylotriose, respectively.

**TABLE 6:** XOSs (mmol/g) production by commercial enzymes at different concentration within 1 hr of incubation

Enzyme concentration (U/g)	10	20	30	40
Pentopan mono BG	X_1_ = 0.60^a^	X_1_ = 0.28^b^	X_1_ = ND	X_1_ = ND
	X_2_ = 0.49^a^	X_2_ = 0.35^b^	X_2_ = 0.22^c^	X_2_ = 0.13^d^
	X_3_ = 0.46^a^	X_3_ = 0.34^b^	X_3_ = 0.10^c^	X_3_ = 0.03^d^
Veron 191	X_1_ = 0.43^a^	X_1_ = 0.23^b^	X_1_ = ND	X_1_ = ND
	X_2_ = 0.65^b^	X_2_ = 0.71^a^	X_2_ = 0.71^a^	X_2_ = 0.50^c^
	X_3_ = ND	X_3_ = 0.02^a^	X_3_ = 0.06^a^	X_3_ = ND

Note

Different superscript alphabets within the same row represent significant difference at *p* ≤ .05.

X1, X2, and X3 represent xylose, xylobiose, and xylotriose, respectively.

According to the datasheet of the enzymes, veron 191 (GH10) and pentopan mono BG (GH11) produce lower DP and higher amounts of DP XOS, respectively. Falk et al. (2014) reported that GH10 xylanases produce a higher amount of short XOS from rye bran than GH11. GH10 xylanases due to a smaller number of binding sites exhibit higher affinities to low DP xylooligosaccharides (shorter XOS), but GH11 xylanases serve as a good choice for the production of DP ≥ 3.

Faryar et al. (2015) reported a conversion factor (CF) of 0.36 (7 hr) for hydrolysis wheat straw xylan to XOS by endoxylanase. CF of xylan (extracted from Sehima nervosum) to XOS (X_2_ + X_3_) was equal to 0.18 within 10.11 hr (Samanta et al., 2012). CF for sugar cane bagasse xylan by a commercial endoxylanase was 0.18 with the most value of it relevant to (X_2_ + X_3_) within 8 hr (Jayapal et al., 2013). Akpinar et al. (2009) evaluated the enzymatic production of XOS from tobacco stalk, sunflower stalk, cotton stalk, and wheat straw, and they reported that CF for tobacco xylan to XOS was about 0.17 which was more than the other treatments. They also reported that the xylanases from *Aspergillus niger* showed the highest activity within 8–24 hr, while the xylanase from *Trichoderma longibrachiatum* showed the highest velocity within the first 8 hr of incubation. The CF for xylan to XOS from garlic's straw by the xylanase from *Bacillus mojavensis* enzyme was 0.29 (Kallel et al., 2015). Chapla et al. (2012) reported that maximum XOS and minimum xylose were obtained within 8–16 hr with 20 U enzyme per g of xylan of corncob, and CF of the hydrolysis was 0.60. Table 5 compares CF and XOS types of various xylans in different studies. Table 7 indicates that the X_2_ (xylobiose) and X_3_ (xylotriose) are the most XOS obtained from various sources. The highest and the lowest CF were observed for corncob (0.6) and different agriculture wastes (0.17), respectively. In this study, CF was 0.36 for veron 191 and 0.41 for pentopan mono BG enzymes. The higher the amount of CF, the more cost‐effective the production of XOS is. On the other hand, the shorter the duration of hydrolysis, the more economical the production of XOS is. The productivity coefficient (PC) is an index for the amount of product at a certain duration. In Table 5, PC of enzymatic production of XOS for xylan sources is presented. Accordingly, the PC for date seed xylan is higher than that for other sources (except of corncob).

**TABLE 7:** Comparison of conversion factor (CF) and XOS type produced from various xylan in different researches

Xylan source	XOS	CF	PC	References
Wheat straw		0.36	0.05	Faryar et al., 2015
Sehima nervosum	Max: X_2_ X_3_	0.18	0.02	Samanta et al., 2012
Sugarcane bagasse	Max: X_2_ X_3_	0.18	0.02	Jayapal et al., 2013
Differential.agri wastes		0.17	0.02	Akpinar et al., 2009
Garlic straw	Max: X_2_ X_3_	0.29	0.04	Kallel et al., 2015
Corncob	X_2_–X_5_	0.17	0.01	Boonchuay et al., 2014
Corncob		0.60	0.08	Chapla et al., 2012
Date seed—veron	X_2_–X_3_	0.36	0.06	This work
Date seed—pentopan	X_2_–X_3_	0.41	0.10	This work

Abbreviations: CF, g XOS/g xylan; PC, CF/hydrolysis time.

## 4 CONCLUSION

The present study was an attempt to identify xylans extracted from date seeds as a new source for XOS production by two traditional xylanases. The results showed that the alkaline pretreatment (0.1 N NaOH at 80°C) is essential prior to xylan extraction from date seed (at room temperature). The type of xylanase, enzyme dosage, and hydrolysis duration had a significant effect on the degree of polymerization and the amount of XOS. The highest XOS production by Pentopan mono BG (1.17 mmol/g) and veron 191 (1.13 mmol/g) observed after 4 and 6 hr of incubation, respectively. Conversion factors of date seed xylan to XOS were 0.41 and 0.36 for pentopan mono BG and veron 191, respectively. Given that XOS had a considerable prebiotic properties, date seed can be suggested as a suitable substrate for prebiotic production.

## CONFLICT OF INTEREST

The authors declare that they have no conflicts of interest.

## ETHICAL APPROVAL

This investigation did not involve human or animal testing.

## Data Availability

The datasets used or analyzed during the current study are available from the corresponding author on reasonable request.
